# Prognostic artificial intelligence model to predict 5 year survival at 1 year after gastric cancer surgery based on nutrition and body morphometry

**DOI:** 10.1002/jcsm.13176

**Published:** 2023-02-12

**Authors:** Heewon Chung, Yousun Ko, In‐Seob Lee, Hoon Hur, Jimi Huh, Sang‐Uk Han, Kyung Won Kim, Jinseok Lee

**Affiliations:** ^1^ Department of Biomedical Engineering, College of Electronics and Information Kyung Hee University Yongin‐si Gyeonggi‐do Republic of Korea; ^2^ Department of Radiology, Asan Medical Center University of Ulsan College of Medicine Seoul Republic of Korea; ^3^ Department of Surgery, Asan Medical Center University of Ulsan College of Medicine Seoul Republic of Korea; ^4^ Department of Surgery Ajou University School of Medicine Suwon Republic of Korea; ^5^ Department of Radiology Ajou University School of Medicine Suwon Republic of Korea

**Keywords:** Gastric cancer, Survival, Prediction, Prognosis, Artificial intelligence

## Abstract

**Background:**

Personalized survival prediction is important in gastric cancer patients after gastrectomy based on large datasets with many variables including time‐varying factors in nutrition and body morphometry. One year after gastrectomy might be the optimal timing to predict long‐term survival because most patients experience significant nutritional change, muscle loss, and postoperative changes in the first year after gastrectomy. We aimed to develop a personalized prognostic artificial intelligence (AI) model to predict 5 year survival at 1 year after gastrectomy.

**Methods:**

From a prospectively built gastric surgery registry from a tertiary hospital, 4025 gastric cancer patients (mean age 56.1 ± 10.9, 36.2% females) treated gastrectomy and survived more than a year were selected. Eighty‐nine variables including clinical and derived time‐varying variables were used as input variables. We proposed a multi‐tree extreme gradient boosting (XGBoost) algorithm, an ensemble AI algorithm based on 100 datasets derived from repeated five‐fold cross‐validation. Internal validation was performed in split datasets (*n* = 1121) by comparing our proposed model and six other AI algorithms. External validation was performed in 590 patients from other hospitals (mean age 55.9 ± 11.2, 37.3% females). We performed a sensitivity analysis to analyse the effect of the nutritional and fat/muscle indices using a leave‐one‐out method.

**Results:**

In the internal validation, our proposed model showed AUROC of 0.8237, which outperformed the other AI algorithms (0.7988–0.8165), 80.00% sensitivity, 72.34% specificity, and 76.17% balanced accuracy. In the external validation, our model showed AUROC of 0.8903, 86.96% sensitivity, 74.60% specificity, and 80.78% balanced accuracy. Sensitivity analysis demonstrated that the nutritional and fat/muscle indices influenced the balanced accuracy by 0.31% and 6.29% in the internal and external validation set, respectively. Our developed AI model was published on a website for personalized survival prediction.

**Conclusions:**

Our proposed AI model provides substantially good performance in predicting 5 year survival at 1 year after gastric cancer surgery. The nutritional and fat/muscle indices contributed to increase the prediction performance of our AI model.

## Introduction

Gastrectomy is a pivotal treatment option which provides the possibility to cure patients with gastric cancer.^1^ Diagnosis at earlier stages, the introduction of perioperative chemotherapy, and advances in surgical techniques have enabled clinicians to achieve better patient survival due to this malignancy.^2^ As the number of long‐term survivors increases, precision medicine and personal stratification of patient prognosis are gaining emphasis because the tumour, node, metastasis (TNM) staging system does not provide accurate predictions of patient survival during and after treatment.^3^

Many prognostic models using various nomograms, scoring systems, and artificial intelligence (AI) models, were developed to predict the overall survival of patients after surgery.^4^, ^5^, ^6^, ^7^ However, none of these models have been used extensively in clinical practices due to the limited accuracy in predicting the survival of patients in various situations.

We hypothesize that one of the main reasons for the inaccuracy is the limited number of prognostic variables available to build an adequate model. For simplicity and the uniform application of the prognostic models, prior models depended on a few known variables such as the TNM staging system, age, sex, tumour location, tumour histology, and the extent of the surgery.^3^, ^6^, ^8^ However, recent studies demonstrated that additional variables could affect patient survival in gastric cancer, such as nutrition, sarcopenia, anaemia, and interval changes in these variables between pre‐operation and post‐operation.^9^, ^10^ Based on these findings, we assumed that a higher accuracy of prognostication could be achieved by an in‐depth analysis of as many variables as possible that could be easily derived from clinical data and body morphometry data derived from imaging.

Based on prior research, we postulated that the optimal timing to predict long‐term survival would be 1 year after gastrectomy when patients are recovered from several changes derived from surgery and adjusted to new metabolic balance.^9^, ^10^ In addition, the time‐varying host factors such as interval change in muscle mass and nutrition between preoperative and postoperative period may influence the long‐term survival, thus should be included in prognostic model.^4^

A deep learning method might be a better tool than conventional prognostic models such as the Cox‐hazard proportional regression model in the construction of a prognostic model that consists of many variables. Deep learning has an advantage in handling big clinical data with non‐linear effects, interactions between variables, and time‐varying effects between variables, such as before and after surgery.^3^ There have been a few encouraging studies in building a prognostic model for patients treated with gastrectomy using deep learning AI techniques^3^, ^7^, ^11^, ^12^; however, there have been shortcomings in the use of the model, which include insufficient patient cohort size, a limited number of variables included in the model, exclusion of non‐disease‐related variables, and inadequate external validation process of the model.

In this study, we aimed to develop and validate an AI prognostic model for predicting the 5 year survival at 1 year after gastric cancer surgery using big data sets (>4000 cases) with many patient‐related variables, including nutrition, skeletal muscle mass, visceral and subcutaneous adipose tissue mass, sarcopenia, obesity, co‐morbidities, and interval changes in these variables between before and after surgery as well as cancer‐related variables.

## Methods

This study was approved by two institutional review boards: Asan Medical Center (AMC), Seoul, Korea (IRB No. 2017‐0216) and Ajou University Hospital (AUH), Suwon, Korea (AJIRB‐MED‐MDB‐22‐012). Informed consent was waived in all participants by IRBs. All methods were performed in accordance with the Transparent Reporting of a multivariable prediction model for Individual Prognosis Or Diagnosis (TRIPOD) guidelines.^13^

### Patient data for AI model development

We used the comprehensive gastric cancer surgery registry that was prospectively built at the AMC between 2003 and 2012. This registry contained data from 6229 patients with 63 clinicopathologic variables. Among the total of 6229 patients, we excluded the deceased within 1 year after surgery or patients with missed follow‐ups (*n* = 688). In addition, we excluded 1516 patients who were missing more than 19 variables (30% of clinical variables) or abdominal CT scans at preoperative period and 1 year after gastrectomy. A total of 4025 patients' data (mean age 56.1 ± 10.9, 36.2% females) from AMC were used in the training of our AI model.

The 63 clinical variables were classified into demographic variables, physical indices, laboratory results, nutritional index, fat/muscle indices, surgery‐related variables, clinic‐pathological information, and co‐morbidities. *Table* 1 summarizes the 63 clinical variables used in creating our AI model and the statistical summaries of the clinical variables in the survived and deceased groups. For the physical index variables, we used height, weight, and body mass index (BMI).

**Table 1:** Clinical variables used for the AI model and statistical summaries of the clinical variables in the survived and deceased groups

No.	Characteristics	Description	Survived	Deceased	*P*‐value
			(*n* = 3849)	(*n* = 176)	
Demographic variables, mean ± SD or *n* (%)					
1	Age at operation (year)		55.70 ± 11.00	64.65 ± 10.39	<0.001
2	Sex	Male	2434 (63.24%)	135 (76.70%)	<0.001
		Female	1415 (36.76%)	41 (23.30%)	
Physical indices, mean ± SD					
3	Height (cm)		163.11 ± 8.15	163.22 ± 7.79	0.8620
4	Preoperative weight (kg)		63.75 ± 10.24	61.31 ± 10.50	0.0020
5	Postoperative 1 year weight (kg)		57.84 ± 9.62	56.39 ± 9.13	0.1409
6	Preoperative BMI (kg/m^2^)		23.90 ± 2.97	22.97 ± 3.28	<0.001
7	1 year postoperative BMI (kg/m^2^)		21.67 ± 2.72	20.98 ± 3.13	0.0141
Laboratory results, mean ± SD					
8	Preoperative cholesterol (mg/dL)		182.95 ± 36.40	168.10 ± 36.88	<0.001
9	1 year postoperative cholesterol (mg/dL)		174.45 ± 33.51	160.42 ± 43.69	<0.001
10	Preoperative haemoglobin (g/dL)		13.51 ± 1.87	12.68 ± 1.99	<0.001
11	1 year postoperative haemoglobin (g/dL)		13.12 ± 1.73	12.18 ± 1.95	<0.001
12	Preoperative albumin (g/dL)		4.03 ± 0.37	3.73 ± 0.45	<0.001
13	1 year postoperative albumin (g/dL)		4.15 ± 0.32	3.76 ± 0.67	<0.001
14	Preoperative protein (g/dL)		6.89 ± 0.53	6.65 ± 0.62	<0.001
15	1 year postoperative protein (g/dL)		7.22 ± 0.46	6.97 ± 0.80	<0.001
Nutritional index, mean ± SD					
16	Preoperative nutritional risk index		102.91 ± 5.68	98.37 ± 6.86	<0.001
17	1 year postoperative nutritional risk index		100.78 ± 6.23	93.06 ± 13.12	<0.001
Body morphometry variables with fat/muscle indices, mean ± SD					
18	Preoperative subcutaneous fat area (cm^2^)		119.61 ± 57.23	104.32 ± 47.21	0.0542
19	1 year postoperative subcutaneous fat area (cm^2^)		80.51 ± 50.12	71.94 ± 49.67	0.1706
20	Preoperative visceral fat area (cm^2^)		98.75 ± 55.09	106.49 ± 65.06	0.3196
21	1 year postoperative visceral fat area (cm^2^)		42.61 ± 35.60	45.84 ± 42.84	0.4737
22	Preoperative skeletal muscle area (cm^2^)		124.77 ± 30.49	119.59 ± 27.79	0.2225
23	1 year postoperative skeletal muscle area (cm^2^)		119.39 ± 27.52	110.40 ± 25.27	0.0087
24	Preoperative skeletal muscle index adjusted with height^2^ (SMA/height^2^, cm^2^/m^2^)		46.54 ± 8.68	45.00 ± 8.40	0.2054
25	1 year postoperative skeletal muscle index adjusted with height^2^ (SMA/height^2^, cm^2^/m^2^)		44.53 ± 7.92	41.57 ± 7.73	0.0028
26	Preoperative skeletal muscle index adjusted with BMI (SMA/BMI)		5.27 ± 1.17	5.17 ± 1.07	0.5570
27	1 year postoperative skeletal muscle index adjusted with BMI (SMA/BMI)		5.50 ± 1.09	5.33 ± 0.98	0.2723
Surgery‐related variables, mean ± SD or *n* (%)					
28	Type of surgery	Total gastrectomy	1002 (26.03%)	59 (33.52%)	0.0016
		Distal gastrectomy	2843 (73.86%)	116 (65.91%)	
		Other gastrectomy	3 (0.08%)	0 (0.00%)	
29	Type of anastomosis	Gastroduodenostomy	2130 (55.34%)	76 (43.18%)	0.0313
		Roux‐en‐Y gastrojejunostomy	235 (6.11%)	8 (4.55%)	
		Gastrojejunostomy without jejunojejunostomy	40 (1.04%)	2 (1.14%)	
		Gastrojejunostomy with jejunojejunostomy	188 (4.88%)	11 (6.25%)	
		Total gastrectomy	884 (22.97%)	53 (30.11%)	
		Others	13 (0.34%)	1 (0.57%)	
30	Intent of treatment	Curative	3840 (99.77%)	168 (95.45%)	<0.001
		Palliative	8 (0.21%)	8 (4.55%)	
31	History of gastric surgery	No	3473 (90.23%)	150 (85.23%)	0.8804
		Yes	27 (0.70%)	1 (0.57%)	
32	History of endoscopic submucosal dissection	No	3368 (87.50%)	146 (82.95%)	0.7584
		Yes	133 (3.46%)	5 (2.84%)	
33	Operation method	Laparoscopy	1480 (38.45%)	36 (20.45%)	<0.001
		Open	2233 (58.02%)	130 (73.86%)	
34	Lymph node dissection	Less than D2	1384 (56.25%)	70 (35.23%)	0.1652
		D2	2301 (11.95%)	93 (11.93%)	
35	Proximal resection margin (cm)		4.52 ± 2.91	4.77 ± 3.09	0.2624
36	Distal resection margin (cm)		7.03 ± 4.91	6.34 ± 5.07	0.0719
Pathologic variables, mean ± SD or *n* (%)					
37	Cancer stage*	Ia	2165 (12.47%)	62 (15.91%)	<0.001
		Ib	460 (7.90%)	21 (9.66%)	
		IIa	480 (5.17%)	28 (6.82%)	
		IIb	304 (4.29%)	17 (11.36%)	
		IIIa	199 (1.84%)	12 (4.55%)	
		IIIb	165 (0.10%)	20 (4.55%)	
		IIIc	71 (35.96%)	8 (39.77%)	
		IV	4 (59.78%)	8 (52.84%)	
38	Number of tumours		1.05 ± 0.21	1.07 ± 0.26	0.1034
39	Tumour size (cm)		4.07 ± 2.62	5.21 ± 3.58	<0.001
40	Number of metastatic lymph nodes		1.14 ± 3.11	3.06 ± 6.73	<0.001
41	Number of retrieved lymph nodes		32.32 ± 13.77	31.71 ± 13.08	0.5675
42	The extranodal extension of the metastatic lymph node (pathological findings)	Negative	684 (17.77%)	38 (21.59%)	0.0412
		Positive	262 (6.81%)	25 (14.20%)	
43	Diameter of the extranodal extension of the metastatic lymph node (mm)		1.63 ± 1.34	2.46 ± 2.00	0.0356
44	Lymphovascular invasion	Negative	2956 (76.80%)	114 (64.77%)	<0.001
		Positive	867 (22.53%)	61 (34.66%)	
45	T stage*	T1	2395 (62.22%)	70 (39.77%)	<0.001
		T2	492 (12.78%)	22 (12.50%)	
		T3	680 (17.67%)	56 (31.82%)	
		T4	276 (7.17%)	27 (15.34%)	
46	N stage*	N0	2884 (74.93%)	110 (62.50%)	<0.001
		N1	430 (11.17%)	16 (9.09%)	
		N2	321 (8.34%)	19 (10.80%)	
		N3	210 (5.46%)	30 (17.05%)	
47	Perineural invasion	Negative	3065 (79.63%)	112 (63.64%)	0.2965
		Positive	701 (18.21%)	58 (32.95%)	
		Not evaluated	15 (0.39%)	1 (0.57%)	
48	Gross appearance of advanced gastric cancer (AGC)	Borrmann type 1	39 (1.01%)	2 (1.14%)	<0.001
		Borrmann type 2	262 (6.81%)	23 (13.07%)	
		Borrmann type 3	921 (23.93%)	63 (35.80%)	
		Borrmann type 4	45 (1.17%)	7 (3.98%)	
		Unclassified	59 (1.53%)	2 (1.14%)	
49	Gross appearance of early gastric cancer (Type 1 to 3)	Type I	95 (2.47%)	2 (1.14%)	<0.001
		Type II	2170 (56.38%)	62 (35.23%)	
		Type III	102 (2.65%)	5 (2.84%)	
50	Tumour histology	Papillary adenocarcinoma	23 (0.60%)	0 (0.00%)	0.0064
		Well‐differentiated tubular adenocarcinoma	389 (10.11%)	13 (7.39%)	
		Moderately‐differentiated tubular adenocarcinoma	956 (24.84%)	58 (32.95%)	
		Poorly‐differentiated tubular adenocarcinoma	1402 (36.43%)	54 (30.68%)	
		Signet‐ring cell carcinoma	576 (14.96%)	16 (9.09%)	
		Mucinous adenocarcinoma	59 (1.53%)	3 (1.70%)	
		Others	60 (1.56%)	3 (1.70%)	
51	Lauren Classification	Intestinal	1615 (41.96%)	84 (47.73%)	0.2198
		Diffuse	1454 (37.78%)	53 (30.11%)	
		Mixed	457 (11.87%)	21 (11.93%)	
		Indeterminate	27 (0.70%)	0 (0.00%)	
52	Tumour location^†^	Upper third	570 (14.81%)	34 (19.32%)	0.1020
53		Middle third	845 (21.95%)	31 (17.61%)	0.1710
54		Lower third	2458 (63.86%)	115 (65.34%)	0.6949
Co‐morbidities, *n* (%)					
55	Diabetes mellitus		384 (9.98%)	35 (19.89%)	<0.001
56	Hypertension		924 (24.01%)	58 (32.95%)	0.0069
57	Chronic viral hepatitis		146 (3.79%)	12 (6.82%)	0.0433
58	Liver cirrhosis		25 (0.65%)	3 (1.70%)	0.0997
59	Tuberculosis		191 (4.96%)	19 (10.80%)	<0.001
60	Myocardial infarction		10 (0.26%)	3 (1.70%)	<0.001
61	Cerebrovascular accident		63 (1.64%)	13 (7.39%)	<0.001
62	Valvular heart disease		5 (0.13%)	0 (0.00%)	0.6324
63	Chronic obstructive pulmonary disease		13 (0.34%)	2 (1.14%)	0.0891
64	Asthma		28 (0.73%)	4 (2.27%)	0.0240
65	Chronic renal failure		6 (0.16%)	2 (1.14%)	0.0043

Abbreviations: SD, standard deviation; BMI, body mass index; SMA, skeletal muscle area.

* Tumour stage according to AJCC Cancer Staging Manual 8th Edition.

† Duplicated count is allowed. For example, we checked all locations for a diffuse Bormann type IV cancer which was in the upper third, middle third, and lower third.

The laboratory results included cholesterol, haemoglobin, albumin, and protein values. For the nutritional index, we used the nutritional risk index (NRI), which was calculated based on the formula ((1.519 × serum albumin g/L) + 0.417 × (present weight/usual weight)) × 100.^14^

For body morphometry data including fat/muscle indices, we used an artificial intelligence solution (AID‐U™, iIAD Inc, Seoul, Korea) to measure subcutaneous fat area (SFA), visceral fat area (VFA) and skeletal muscle area (SMA) at L3 vertebral body level on abdominal CT scans. The skeletal muscle mass index (SMI) was calculated by the SMA divided by the height squared (SMA/height^2^) or by the adjusted body mass index (SMA/BMI).^15^

For the surgical information, we used the type of gastrectomy, the type of anastomosis, treatment intention, history of previous gastric surgery or endoscopic submucosal dissection (ESD), operation method (open vs. laparoscopic approach), extent of lymph node dissection, and length of proximal and distal resection margins.

For clinicopathological data, we used cancer stage, number of tumours, tumour size, number of metastatic lymph nodes, number of retrieved lymph nodes, the presence and diameter of extranodal extension of a metastatic lymph node, the diameter of an extranodal extension of a metastatic lymph node, the presence of lymphovascular invasion and perineural invasion by tumour cells, gross appearance of advanced gastric cancer and early gastric cancer, pathological tumour type, Lauren classification, and tumour location.^16^, ^17^, ^18^

For co‐morbidities, we included diabetes mellitus, hypertension, chronic active hepatitis, liver cirrhosis, tuberculosis, myocardial infarction, cerebrovascular accidents, valvular heart disease, chronic obstructive pulmonary disease, asthma, and chronic renal failure in the input variables.

The effects of interval changes in variables between preoperative and 1 year postoperative records were recorded as time‐varying measurements. Changes in physical indices, laboratory results, the nutritional index, and fat/muscle indices, specifically weight, BMI, cholesterol, haemoglobin, albumin, protein, NRI, SFA, SMA, SMI, and SMA/BMI, were calculated as the time‐varying indices.

### Patient data for external validation

A total of 590 patients' data (mean age 55.9 ± 11.2, 37.3% females) were used as an external validation for our AI model. The data was collected based on a comprehensive gastric cancer registry prospectively built at the AUH between 2010 and 2012. In the AUH dataset, only 28 of the 63 clinical variables were available. *Table* 2 summarizes the available clinical variables from the AUH dataset. The AUH's clinical variables did not include all the co‐morbidities. Only the cancer stage was considered in the clinic‐pathological information. The surgical information only included the types of surgery and anastomosis. The other clinical variables such as age/sex, physical indices, laboratory results, and body morphometry data with fat/muscle indices were all measured, except for protein values (*Table* S1). It was challenging but worthwhile to evaluate the developed AI model only using a fraction of the clinical variables, which mostly were age/sex, physical indices, laboratory information, and fat/muscle indices.

**Table 2:** Clinical variables used for external validation of our AI model

No.	Clinical variables		No.	Clinical variables	
Demographic variables			20	Preoperative skeletal muscle area (cm^2^)	
1	Age at operation (year)		21	1 year postoperative skeletal muscle area (cm^2^)	
2	Sex	Male	22	Preoperative skeletal muscle index (cm^2^/m^2^)	
		Female	23	1 year postoperative skeletal muscle index (cm^2^/m^2^)	
Physical indices			24	Preoperative skeletal muscle index	
3	Height (cm)		25	1 year postoperative skeletal muscle index	
4	Preoperative weight (kg)		Surgery‐related variables		
5	1 year postoperative weight (kg)		26	Type of surgery	Total gastrectomy
6	Preoperative BMI				Distal gastrectomy
7	1 year postoperative BMI				Other gastrectomy
Laboratory results			27	Type of anastomosis	Gastroduodenostomy
8	Preoperative cholesterol (mg/dL)				Roux‐en‐Y gastrojejunostomy
9	1 year postoperative cholesterol (mg/dL)				Gastrojejunostomy without jejunojejunostomy
10	Preoperative haemoglobin (g/dL)				Gastrojejunostomy with jejunojejunostomy
11	1 year postoperative haemoglobin (g/dL)				Total gastrectomy
12	Preoperative albumin (g/dL)				Others
13	1 year postoperative albumin (g/dL)		Pathologic variables		
Nutritional index			28	Cancer stage	Ia
14	Preoperative nutritional risk index				Ib
15	1 year postoperative nutritional risk index				IIa
Body morphometry variables with fat/muscle indices					IIb
16	Preoperative subcutaneous fat area (cm^2^)				IIIa
17	1 year postoperative subcutaneous fat area (cm^2^)				IIIb
18	Preoperative visceral fat area (cm^2^)				IIIc
19	1 year postoperative visceral fat area (cm^2^)				IV

### Final variables with derived time‐varying variables

For the AI model to predict the 5 year survival, 63 clinical variables were used. In addition to the 63 variables, the differences in values and percentages between the preoperative and 1 year postoperative weight, BMI, cholesterol, haemoglobin, albumin, protein, NRI, SFA, SMA, SMI, and SMA/BMI were considered. The difference in values was calculated by subtracting the 1 year postoperative measurement from the preoperative value. The percent difference was calculated by dividing the difference in values by the preoperative value. Using the difference in values, the percent difference in values, and the one‐hot encoded categorical values, 26 new variables were derived. Thus, a total of 89 variables were used for the AI model (*Table* S2). The external validation data had 28 variables available, which was extended to 54 variables (*Table* S2).

### Data split and cross‐validation

In this study, our data is composed of training, internal validation, and external validation data. The AMC data was split into training and internal validation data with a ratio of 8:2 in a stratified fashion. The internal validation dataset was used only for an independent test of the developed AI model and not for training. In addition, the whole AUH dataset was used only for external validation and never used in the model training. *Table* S3 summarizes the datasets for training, internal validation, and external validation.

A five‐fold cross‐validation was performed and repeated 20 times to confirm the model's generalization ability using the training data. The training dataset (*n* = 3220) was first randomly shuffled and divided into five equal groups in a stratified manner. Subsequently, four groups were selected for training the model, and the remaining group was used for testing. This process was repeated five times by changing which group was the testing data. The whole process was repeated 20 times. The finalized AI model was based on the repeated five‐fold cross‐validation and is described in subsequent sections. We evaluated the performance of the AI model using the internal validation data and the external validation data.

### Preprocessing

There were missing variables in the AMC (training and internal validation data) and AUH (external validation data) datasets (*Table* S4). The average and standard deviations of the missing data in the AMC and AUH datasets were 26.7 ± 30.4%, and 0.1 ± 0.1%, respectively. Note that the percentages of missing data for AUH were considered only for the available variables summarized in *Table* 2. The missing variable in the training data was replaced with the missing variable's mean from across the training, internal validation, and external validation datasets. The same variable replacement method was also applied to replace the unavailable variables in the AUH dataset.

A dataset standardization was performed and is a common requirement for machine learning estimators. The standardization changes the data distribution of each variable with a mean of zero and a standard deviation of one using the equation:

(1) $${\text{Data}}_{\text{stand}a\mathrm{rd}}=\frac{\text{Data}-\text{mean}\left(\text{train}\right)}{\mathrm{SD}\left(\text{train}\right)}\;,$$

where $\text{mean}\left(\text{train}\right)$ and $\mathrm{SD}\left(\text{train}\right)$ are the mean and standard deviation of each variable in the training dataset. The standardization was applied to the training, internal validation, and external validation datasets.

### Multi‐tree extreme gradient boosting

The extreme gradient boosting (XGBoost) model was adopted to develop the AI model to predict the 5 year survival.^19^ In this study, we trained the XGBoost model using the five‐fold cross‐validation method and repeated this 20 times using the training data. Then, the results were ensembled into 100 trees with soft voting. Figure S1 illustrates the ensemble AI model, based on the combination of the 100 trees produced by the XGBoost algorithms. Each tree was produced from the XGBoost algorithm by setting the maximum depth to 2, the learning rate to 0.1, the number of tree estimators to 50, the value of the regularization parameter *α* to 0.8, the fraction of observations to 0.2, and the fraction of columns to 0.8.

In this study, the number of deceased patients was much lower than the number of survived patients. Thus, for each tree from XGBoost, we up‐sampled the deceased patient data using the synthetic minority over‐sampling technique (SMOTE), aiming to prevent the model's bias toward the survived patient data by balancing the data in the two groups. After modelling the multi‐tree XGBoost model, the contribution of each of the 89 variables to the prediction of survival was investigated via a variable importance analysis. The repeated five‐fold cross‐validation provided 100 sets of important variables. We then averaged and normalized the sets of important variables in order that the values from each classifier were in the range from zero to one.

To compare the performance of our proposed predictive AI model, we separately trained the following models, random forest (RF),^20^ gradient boosting machine (GBM),^21^ adaptive boosting (Adaboost),^22^, ^23^ light gradient boosting machine (LightGBM),^24^ categorical boosting (CatBoost),^25^ and ensemble models including XGBoost.^19^ The implementation and analysation of the machine learning models was done using Imbalanced‐learn (version 0.8.1), NumPy (version 1.17), Scikit‐learn (version 0.24.2), Pandas (version 0.24.2), Matplotlib (version 3.1.0), XGBoost (version 0.90), and LightGBM (version 3.3.0).

### Performance evaluation of AI models

The performance of our AI model's prediction was evaluated and compared based on repeated K‐fold cross‐validation using the isolated testing data. The model was additionally evaluated with the external validation data. Sensitivity, specificity, accuracy, and balanced accuracy metrics were evaluated and defined as:

(2) $$\text{Sensitivity}=\frac{\mathrm{TP}}{\mathrm{TP}+\mathrm{FN}\;},$$

(3) $$\text{Specificity}=\frac{\mathrm{TN}}{\mathrm{TN}+\mathrm{FP}\;},$$

(4) $$\text{Accuracy}=\frac{\mathrm{TP}+\mathrm{TN}}{\mathrm{TP}+\mathrm{TN}+\mathrm{FP}+\mathrm{FN}\;},$$

(5) $$\text{Balanced Accuracy}=\frac{\text{Sensitivity}+\text{Specificity}}{2\;},$$

True positive (TP), true negative (TN), false positive (FP), and false negative (FN) were used in the evaluations. The balanced accuracy evaluated the imbalance between the survived and the deceased groups. In addition, we computed the area under the receiver operating characteristics (AUROC).

### Sensitivity analysis

In addition to developing an AI model, we included variables corresponding to pre‐ and post‐operative nutritional and body morphometry data such as NRI, SFA, VFA, SMA, SMI, and SMA/BMI. To investigate the effect of these variables, we adopted a leave‐one‐out method by excluding the pre‐ and post‐operative nutritional and fat/muscle indices from the variables and repeated the training of the AI model. Subsequently, we evaluated the prediction performance based on cross‐validation, internal validation, and external validation data.

### Public website deployment

We deployed the AI model on a public web server (http://ai‐research.co.kr/survival) through Amazon Web Services, which provides a secure, durable, and scalable service. After accessing the website, a user enters the clinical variables, which are encoded by the website's server, and users can immediately obtain the predicted 5 year survival. There is no need to enter private information other than the clinical variables. The entered information is immediately deleted after the prediction is derived, so there is no risk of information exposure. Code is available at https://github.com/HeewonChung92/Gastric_Cancer_Survival.

## Results

### Variable importance rankings

*Table* 3 shows the ranked averages of the important variables using XGBoost via the repeated K‐fold cross‐validation method. The results showed that 25 variables contributed to the prediction of survival. Among the variables, age had the highest importance value, followed by preoperative albumin, preoperative NRI, T stage, and the percent difference of haemoglobin. In addition, the results showed that co‐morbidities and surgical information did not contribute to the prediction of survival. Furthermore, among the top 25 variables, 15 were related to the pre‐and postoperative variables, including physical indices, laboratory results, the nutritional index, and fat/muscle indices. These pre‐ and postoperative variables indicated that the patient's physical, nutritional, fat/muscle status, and laboratory results before and after the gastric cancer surgery were important variables in predicting the survival of the patients. In addition, it should be noted that the 1 year postoperative NRI, SMI, VFA, and the difference in NRI were included in the top 25 variables even though the percentage of missing data was high: 53.9% for NRI, 69.9% for SMI, 69.8% for VFA, and 53.9% for the difference in NRI.

**Table 3:** Variable importance and percentage of missing data

Variable name	XGBoost	Missing data (%)	Variable name	XGBoost	Missing data (%)
Age	1.0000	0.00	1 year postoperative protein	0.3461	3.43
Preoperative albumin	0.7522	0.00	Postoperative 1 year cholesterol	0.3358	3.40
Preoperative NRI	0.6870	0.05	Perineural invasion‐negative	0.3167	1.84
T stage	0.5638	0.17	Early gastric cancer II	0.2919	39.50
Difference in the percentage of haemoglobin	0.4594	1.96	Diameter of the extranodal extension of the metastatic lymph node	0.2509	96.20
Cancer stage	0.4536	0.02	Preoperative weight	0.2360	0.05
1 year postoperative NRI	0.4533	53.91	Difference in the percentage of albumin	0.2351	3.43
Tumour size	0.4228	0.15	Preoperative BMI	0.2347	0.20
Number of metastatic lymph nodes	0.4087	0.12	Difference in albumin	0.2328	3.43
Difference in haemoglobin	0.3988	1.96	Perineural invasion‐positive	0.2142	1.84
1 year postoperative SMI	0.3873	69.89	Sex	0.2007	0.00
1 year postoperative VFA	0.3843	69.84	Difference in nutritional risk index	0.0476	53.91
1 year postoperative albumin	0.3545	3.43			

### K‐fold cross‐validation results

Based on the repeated five‐fold cross‐validation, the AI model shows a sensitivity of 76.77%, a specificity of 75.26%, an accuracy of 75.32%, a balanced accuracy of 76.01%, and an AUROC of 0.8118 (*Table* 4). The results showed that the AI model provided higher values of sensitivity, specificity, accuracy, balanced accuracy, and AUROC than those from any other models, including RF, GBM, AdaBoost, LightGBM, CatBoost, and Ensemble.

**Table 4:** Repeated K‐fold cross‐validation results in comparison with other machine learning algorithms (mean ± standard deviation)

Cross‐validation results (*n* = 4025)					
Model	Sensitivity	Specificity	Accuracy	Balanced Accuracy	AUROC
RF	0.7451 ± 0.1164	0.7079 ± 0.1152	0.7096 ± 0.1059	0.7265 ± 0.0313	0.7741 ± 0.0348
GBM	0.7531 ± 0.1180	0.7266 ± 0.1122	0.7276 ± 0.1032	0.7398 ± 0.0322	0.7846 ± 0.0355
AdaBoost	0.7140 ± 0.1188	0.7475 ± 0.1190	0.7462 ± 0.1100	0.7307 ± 0.0400	0.7844 ± 0.0435
LightGBM	0.7545 ± 0.1302	0.7197 ± 0.1302	0.7213 ± 0.1200	0.7371 ± 0.0371	0.7846 ± 0.0442
CatBoost	0.7446 ± 0.1045	0.7355 ± 0.0944	0.7359 ± 0.0865	0.7401 ± 0.0334	0.7932 ± 0.0376
Ensemble	0.7550 ± 0.1213	0.7242 ± 0.1200	0.7256 ± 0.1104	0.7396 ± 0.0334	0.7921 ± 0.0361
XGBoost	0.7677 ± 0.0689	0.7526 ± 0.0678	0.7532 ± 0.0622	0.7601 ± 0.0159	0.8118 ± 0.0147

Abbreviations: RF, random forest; GBM, gradient boosting machine; AdaBoost, adaptive boosting; LightGBM, light gradient boosting machine; CatBoost, categorical boosting.

### Internal validation results

Using the isolated split data (*n* = 1121) only for internal validation, the AI model showed a sensitivity of 80.00%, a specificity of 72.34%, an accuracy of 72.67%, a balanced accuracy of 76.17%, and an AUROC of 0.8237. *Table* 5 summarizes the internal validation data results in comparison with other machine learning algorithms. The results showed that our AI model provided higher values of balanced accuracy and AUROC than those from any other model.

**Table 5:** Internal validation results in comparison with other machine learning algorithms

Model	Sensitivity	Specificity	Accuracy	Balanced accuracy	AUROC
RF	0.7429	0.7130	0.7143	0.7279	0.8023
GBM	0.7714	0.7416	0.7429	0.7565	0.8165
AdaBoost	0.7143	0.7455	0.7441	0.7299	0.7988
LightGBM	0.7714	0.7325	0.7342	0.7519	0.8140
CatBoost	0.7143	0.7909	0.7876	0.7526	0.8024
Ensemble	0.7143	0.7857	0.7826	0.7500	0.8160
Ensemble multi‐tree XGBoost	0.8000	0.7234	0.7267	0.7617	0.8237

Abbreviations: RF, random forest; GBM, gradient boosting machine; AdaBoost, adaptive boosting; LightGBM, light gradient boosting machine; CatBoost, categorical boosting.

### External validation results

With the independent external validation data (*n* = 590), our AI model showed a sensitivity of 86.96%, a specificity of 74.60%, an accuracy of 75.08%, a balanced accuracy of 80.78%, and an AUROC of 0.8903 (*Table* 6). The overall accuracy increased with the external validation data compared with the internal validation data with a change in sensitivity from 80.00% to 86.96%, specificity from 72.34% to 74.60%, accuracy from 72.67% to 75.08%, balanced accuracy from 76.17% to 80.78%, and AUROC from 0.8237 to 0.8903, even though the external validation data only had 58 extended variables (28 original variables), compared with a total of 89 variables included in training the model.

**Table 6:** External dataset results

Model	TN	FP	FN	TP	Sensitivity	Specificity	Accuracy	Balanced accuracy	AUROC
Ensemble multi‐tree XGBoost	423	144	3	20	0.8696	0.7460	0.7508	0.8078	0.8903

Abbreviations: TN, true negative; FP, false positives; FN, false negatives; TP, true positives.

Although there were fewer variables in the external validation data, there were enough variables with high importance values. Among the top 25 variables in the training dataset, the external validation dataset included 17 variables. Specifically, the external validation data included six variables among the top seven predictive variables, including age, preoperative albumin, preoperative NRI, the difference in the percentage of haemoglobin, cancer stage, and 1 year postoperative NRI. The external validation dataset had less pre‐ and postoperative missing data than the internal validation dataset. More specifically, among the top 25 variables in the training and internal validation datasets, the percentages of missing 1 year postoperative data for NRI, SMI, VFA, and the difference in NRI were 53.9%, 69.9%, 69.8%, and 53.9%, respectively. On the other hand, in the external validation dataset, the percentages of missing 1 year postoperative data for NRI, SMI, VFA, and the difference in NRI were only 0.2%, 0.0%, 0.0%, and 0.2%, respectively. The lower number of missing data in the pre‐ and postoperative variables improved the predictive model's performance.

### Sensitivity analysis for effect of the nutritional and fat/muscle indices

*Table* 7 summarizes the sensitivity analysis results from this new AI model trained without the nutritional and fat/muscle indices in comparison with our AI model. The difference (diff) represents the accuracy metrics value from our AI model subtracted from the new model without nutritional and fat/muscle indices. The results showed that the balanced accuracy and AUROC decreased by 1.70% and 0.0223, respectively, using the cross‐validation dataset. Using the internal validation dataset, the balanced accuracy and AUROC decreased by 0.31% and 0.0076, respectively. Using the external validation dataset, the balanced accuracy and AUROC decreased by 6.29% and 0.0213, respectively. These results indicated that the nutritional and fat/muscle indices improved the prediction performance of our AI model.

**Table 7:** Performance summarization when nutritional and fat/muscle indices are excluded

Datasets	Sensitivity		Specificity		Accuracy		Balanced accuracy		AUROC	
	value	diff*	value	diff	value	diff	value	diff	value	diff
CV	0.7861	−0.0184	0.7002	−0.0524	0.7041	−0.0491	0.7431	−0.0170	0.7895	−0.0223
Internal validation data	0.8000	0.0000	0.7173	−0.0061	0.7204	−0.0063	0.7586	−0.0031	0.8161	−0.0076
External validation data	0.7826	−0.0870	0.7072	−0.0388	0.7102	−0.0406	0.7449	−0.0629	0.8690	−0.0213

* diff: the value from our AI model minus the value from the model without nutritional and fat/muscle indices.

Abbreviations: AUROC, area under the receiver operating characteristics; CV, cross‐validation.

### Website deployment

**Figure 1:**
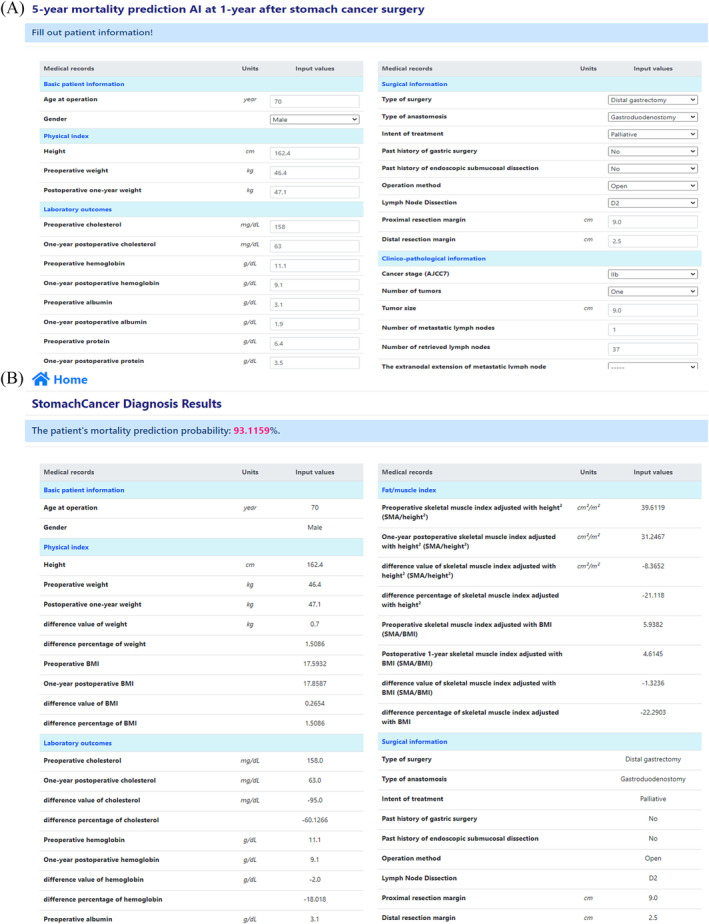
Our AI model on the public website (http://ai‐research.co.kr/survival)

The web application provides the 5 year survival probability at 1 year after gastric surgery, as shown in Figure 1. A user inputs quantified 89 clinical variables (Figure 1(A)), and the user is provided with a 5 year survival prediction (Figure 1(B)).

## Discussion

Our 5 year predictive AI model, multi‐tree XGBoost, was able to predict 5 year survival at 1 year after gastric surgery with high accuracy with data from two medical institutions. The internal validation dataset, data from AMC, had a balanced accuracy of 76.17% and an AUROC of 0.8237. The external validation dataset, data from AHU, had a balanced accuracy of 80.78% and an AUROC of 0.8903.

We complied our study datasets to have several unique characteristics. First, we incorporated as many variables in the model as possible, initially 63 variables, to reflect modern clinical practices and patient characteristics. Second, we incorporated variables that exceeded routine clinicopathological data, including nutritional and fat/muscle indices, such as NRI, SFA, VFA, SMA, SMI, and SMA/BMI. Third, both preoperative and postoperative variables were included in our model to reflect time‐varying effects in variables. Simply using preoperative or postoperative variables alone might not reflect the physiological changes from gastrectomy. Finally, we trained our AI model using large, well‐curated datasets. Thus, data from 4025 patients were included from high‐volume specialty centres. To the best of our knowledge, this study used the largest cohort for an AI predictive model for patients with gastric cancer treated with gastrectomy; this enabled us to build an AI model with high prediction accuracy.

In this study, we adopted the Ensemble XGBoost model. The XGBoost model is a highly recognized machine learning approach for its efficiency and accuracy, and as one of the boosting algorithms, it integrates multiple tree models and delivers an improved prediction accuracy. We applied ensemble learning with a soft voting algorithm to enhance prediction accuracy. We postulated that our technical approach was well suited for an AI prediction model with many variables. The prediction accuracy of our AI model compared with other AI algorithms, including RF, GBM, AdaBoost, LightGBM, CatBoost, and Ensemble, our Ensemble XGBoost model yielded the highest prediction accuracy.

Interestingly, part of our study results demonstrated the effect of nutritional and fat/muscle indices on patient survival using the leave‐one‐out sensitivity analysis method. When we excluded each of the pre‐ and postoperative nutritional and fat/muscle indices, the prediction accuracy decreased in both the internal validation dataset and external validation dataset. In addition, the feature importance analysis showed that most nutritional and fat/muscle indices were the relatively high contributors in predicting the 5 year survival: preoperative NRI (3rd), 1 year postoperative NRI (7th), 1 year postoperative SMI (11th), 1 year postoperative VFA (12th), 1 year postoperative protein (14th), postoperative 1 year cholesterol (15th), preoperative weight (19th), preoperative BMI (21th) and difference in nutritional risk index (25th) among the 64 variables. These results clearly showed that incorporating the nutritional and fat/muscle indices improved the prediction performances of the AI model. Previous studies reported that prolonged malnutrition early in life increased the risk of gastric cancer mortality later in life.^26^ The majority of patients with gastric cancer experienced cancer anorexia‐cachexia syndrome with weight loss, reduced appetite, fatigue, and weakness.^27^ In addition, it was reported that malnutrition before and after gastrectomy significantly and adversely affected overall survival.^28^ Our study is the first to use the pre‐ and postoperative nutritional and fat/muscle indices in machine learning algorithms, whereas previous studies have only performed an analysis based on patient statistics.^26^, ^27^, ^28^, ^29^, ^30^, ^31^

The main causes of post‐gastrectomy death vary along the time after gastrectomy.^32^ Of these, our AI model is intended to predict long‐term survival at 1 year after gastrectomy using big data sets including patients' status of nutrition and body morphometry. Our AI model does not intend to predict early post‐gastrectomy mortality which is mainly contributed to old age, metabolic/nutritional imbalance, tumour recurrence, and postoperative complications.^33^, ^34^ Therefore, we excluded all patients who deceased within 1 year after gastrectomy and did not include postoperative complications in our model.

In our AI model, we did not include the surgeon factors such as extent of experience and surgical skill level. As a high‐volume centre, we perform about 1800 gastrectomy per year. We have put huge efforts to standardize the operation procedures as well as patient management processes, as reported previously.^35^ Thus, we did not consider the surgeon factor in our AI model.

Our model is available as a web toolkit, so anyone can use our AI model. Currently, the application does not store any information entered by users. However, we plan to store information entered by users upon agreement to improve the AI model via a real‐time learning process. We will use our developed web application to acquire additional data and perform real‐time training to update the model.

Though our AI model demonstrated high accuracy in predicting the 5 year survival, several limitations exist. First, our AI model was trained using data from a single high‐volume specialty gastrectomy centre. Because our institution performs approximately 1800 gastric surgeries every year, our patients' survival might exceed those of other institutions or clinical researchers. In addition, we validated our AI model using a single external institution that is also a high‐volume specialty gastrectomy centre. Next, our data included only Korean patients. In future studies, we will train and apply our AI model to more datasets comprising more diverse subjects. To overcome these generalization issues, it may be necessary to validate our AI model using external datasets, such as data from various medical institutions. In addition, we plan to further develop our AI model using extended variables. Finally, the training dataset had a high percentage of missing. Notably, most pre‐ and postoperative variables corresponding to nutritional and fat/muscle indices had 70% or higher missing data. Nevertheless, we achieved high accuracy which might be attributed to the imputation method to replace missing values in the training dataset. In general, accuracy is higher on imputed dataset as compared with incomplete dataset.^36^ In the future, it is important to collect more data with as much information as possible.

In conclusion, we developed an AI model to predict the 5 year survival probability in gastric cancer patients treated with gastrectomy 1 year after surgery using a large training cohort and many variables, including pre‐ and postoperative nutritional and fat/muscle indices. Our performance in predicting 5 year survival is overall accurate and may be helpful for healthcare providers and patients to increase survival after gastrectomy.

## Conflict of interest

The authors declare no competing interests.

## Supporting information


**Figure S1.** Scheme of the multi‐tree XGBoost ensemble AI Model.

**Table S1.** Statistical summary of clinical features of external validation dataset (AHU dataset)
**Table S2.** The final 114 feature list including the extended features, where the bold fonts indicate the extended features.
**Table S3.** Dataset summaries for training, internal validation, and external validation.
**Table S4.** Missing data rates for each feature according to the survived and deceased groups.
