# Supplementary material for: Prognostic artificial intelligence model to predict 5 year survival at 1 year after gastric cancer surgery based on nutrition and body morphometry
Source: J Cachexia Sarcopenia Muscle. 2023 Feb 12;14(2):847–59. doi: 10.1002/jcsm.13176 (PMC10067496; doi:10.1002/jcsm.13176)
Supplement: Supplementary file 2 — Table S1. Statistical summary of clinical features of external validation dataset (AHU dataset) Table S2. The final 114 feature list including the extended features, where the bold fonts indicate the extended features. Table S3. Dataset summaries for training, internal validation, and external validation. Table S4. Missing data rates for each feature according to the survived and deceased groups. [file JCSM-14-847-s001.docx]

**Prognostic AI Model to predict Five-year survival at One-Year after Gastric cancer Surgery based on nutrition and body morphometry**

# ^1^Heewon Chung^‡^, ^2^Yousun Ko^‡^, ^3^In-Seob Lee^‡^, ^4^ Hoon Hur, ^5^Jimi Huh, ^4^Sang-Uk Han, ^3,6^KyungWon Kim*, and ^1^Jinseok Lee*

^1^Department of Biomedical Engineering, College of Electronics and Information, Kyung Hee University, Yongin-si, Gyeonggi-do, 17104, Republic of Korea

^2^Department of Surgery, Asan Medical Center, University of Ulsan College of Medicine, Seoul, 05505, Republic of Korea

^3^Biomedical Research Center, Asan Institute for Life Sciences, Asan Medical Center, Seoul, 05505, Republic of Korea

^4^Department of Surgery, Ajou University School of Medicine, Suwon, 16499, Republic of Korea

^5^Department of Radiology, Ajou University School of Medicine, Suwon, 16499, Republic of Korea

^6^Department of Radiology, Asan Medical Center, University of Ulsan College of Medicine, Seoul 05505, Republic of Korea

^‡^ These authors contributed equally to this work as first authors.

*These authors contributed equally to this work as corresponding authors.

Corresponding author: Kyung Won Kim (e-mail: medimash@gmail.com) and Jinseok Lee (e-mail:  gonasago@khu.ac.kr).


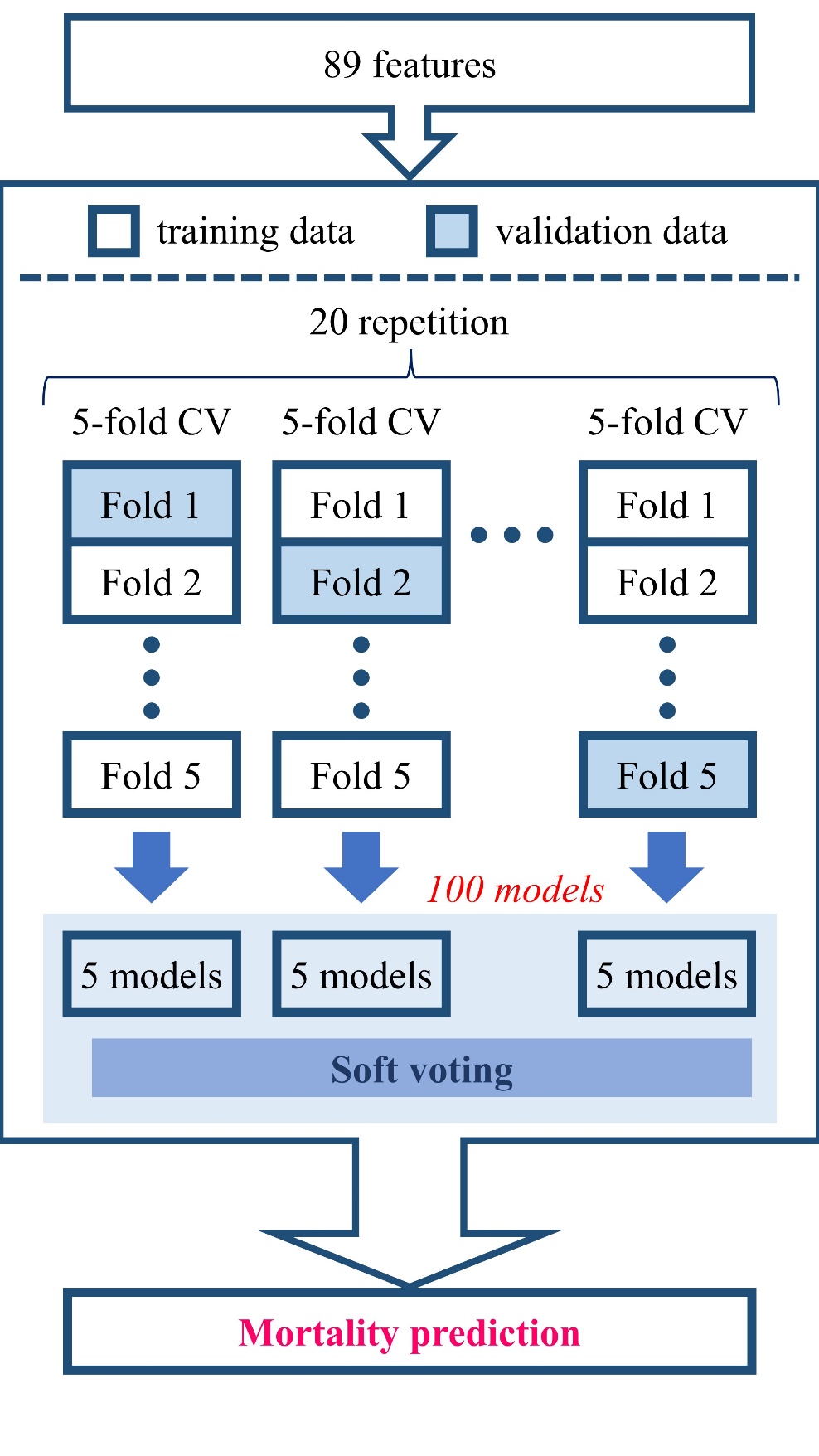


**Figure S1.** Scheme of the multi-tree XGBoost ensemble AI Model.

**Table S1.** Statistical summary of clinical features of external validation dataset (AHU dataset).

| **No** | **Characteristics** | **Description** | **Survived** | **Deceased** | ***p*-value** |
| --- | --- | --- | --- | --- | --- |
|  |  |  | (*n*=567) | (*n*=23) |  |
| **Demographic variables, mean ± SD or n (%)** | | | | | |
| 1 | Age at operation (year) | | 55.50 ± 11.34 | 65.48 ± 9.62 | < 0.001 |
| 2 | Gender | Male | 351 (61.90%) | 19 (82.61%) | 0.0442 |
|  |  | Female | 216 (38.10%) | 4 (17.39%) |  |
| **Physical indices, mean ± SD** | | | | | |
| 3 | Height (cm) | | 163.79 ± 7.90 | 164.82 ± 7.82 | 0.5373 |
| 4 | Preoperative weight (kg) | | 64.35 ± 10.93 | 61.47 ± 12.56 | 0.2192 |
| 5 | Postoperative one-year weight (kg) | | 57.36 ± 9.48 | 52.66 ± 9.53 | 0.0202 |
| 6 | Preoperative BMI | | 23.91 ± 3.15 | 22.53 ± 3.70 | 0.0421 |
| 7 | Postoperative one-year BMI (kg) | | 21.30 ± 2.63 | 19.32 ± 2.74 | < 0.001 |
| **Laboratory results, mean ± SD** | | | | | |
| 8 | Preoperative cholesterol (mg/dL) | | 181.82 ± 37.32 | 161.74 ± 41.57 | 0.0121 |
| 9 | Postoperative one-year cholesterol (mg/dL) | | 170.77 ± 30.30 | 159.91 ± 37.84 | 0.0960 |
| 10 | Preoperative hemoglobin (g/dL) | | 13.65 ± 1.78 | 12.36 ± 2.54 | < 0.001 |
| 11 | Postoperative one-year hemoglobin (g/dL) | | 12.86 ± 1.77 | 12.12 ± 1.77 | 0.0500 |
| 12 | Preoperative albumin (g/dL) | | 4.35 ± 0.36 | 3.98 ± 0.50 | < 0.001 |
| 13 | Postoperative one-year albumin (g/dL) | | 4.36 ± 0.27 | 3.96 ± 0.61 | < 0.001 |
| **Nutritional index, mean ± SD** | | | | | |
| 14 | Preoperative nutritional risk index | | 107.82 ± 5.49 | 102.20 ± 7.62 | < 0.001 |
| 15 | Postoperative one-year nutritional risk index | | 103.49 ± 5.29 | 96.26 ± 10.40 | < 0.001 |
| **Body morphometry variables with fat/muscle indices, mean ± SD** | | | | | |
| 16 | Preoperative subcutaneous fat area (cm^2^) | | 127.04 ± 57.49 | 100.92 ± 46.77 | 0.0320 |
| 17 | Postoperative one-year subcutaneous fat area (cm^2^) | | 85.44 ± 50.06 | 56.13 ± 38.46 | 0.0057 |
| 18 | Preoperative visceral fat area (cm^2^) | | 114.77 ± 69.16 | 114.37 ± 89.44 | 0.9785 |
| 19 | Postoperative one-year visceral fat area (cm^2^) | | 50.84 ± 41.25 | 45.12 ± 49.23 | 0.5181 |
| 20 | Preoperative skeletal muscle area (cm^2^) | | 132.90 ± 29.76 | 126.65 ± 26.09 | 0.3218 |
| 21 | Postoperative one-year skeletal muscle area (cm^2^) | | 126.10 ± 27.58 | 112.43 ± 20.81 | 0.0191 |
| 22 | Preoperative skeletal muscle index (cm^2^/m^2^) | | 49.13 ± 8.48 | 46.30 ± 7.28 | 0.1148 |
| 23 | Postoperative one-year skeletal muscle index (cm^2^/m^2^) | | 46.65 ± 8.00 | 41.24 ± 6.25 | 0.0014 |
| 24 | Preoperative skeletal muscle index | | 5.57 ± 1.07 | 5.67 ± 1.05 | 0.6768 |
| 25 | Postoperative one-year skeletal muscle index | | 5.93 ± 1.10 | 5.86 ± 1.01 | 0.7748 |
| **Surgery-related factors, mean ± SD or n (%)** | | | | | |
| 26 | Type of surgery | Total gastrectomy | 119 (3.09%) | 6 (3.41%) | 0.5582 |
|  |  | Distal gastrectomy | 448 (11.64%) | 17 (9.66%) | 0.5582 |
|  |  | Other gastrectomy | 0 (0.00%) | 0 (0.00%) | - |
| 27 | Type of anastomosis | Gastroduodenostomy | 171 (30.16%) | 9 (39.13%) | 0.3605 |
|  |  | Roux-en-Y gastrojejunostomy | 81 (14.29%) | 3 (13.04%) | 0.8675 |
|  |  | Gastrojejunostomy without jejunojejunostomy | 171 (30.16%) | 4 (17.39%) | 0.1894 |
|  |  | Gastrojejunostomy with jejunojejunostomy | 21 (3.70%) | 1 (4.35%) | 0.8733 |
|  |  | Total gastrectomy | 118 (20.81%) | 6 (26.09%) | 0.5435 |
|  |  | Others | 5 (0.13%) | 0 (0.00%) | 0.6517 |
| **Pathologic variables, mean ± SD or n (%)** | | | | | |
| 28 | Cancer stage^*^ | Ia | 330 (58.20%) | 9 (39.13%) | < 0.001 |
|  |  | Ib | 80 (14.11%) | 4 (17.39%) | < 0.001 |
|  |  | IIa | 67 (11.82%) | 0 (0.00%) | - |
|  |  | IIb | 32 (5.64%) | 1 (4.35%) | 0.1291 |
|  |  | IIIa | 20 (3.53%) | 1 (4.35%) | 0.0672 |
|  |  | IIIb | 26 (4.59%) | 3 (13.04%) | 0.0013 |
|  |  | IIIc | 8 (1.41%) | 5 (21.74%) | < 0.001 |
|  |  | IV | 4 (0.71%) | 0 (0.00%) | - |

** according to AJCC Cancer Staging Manual 8th Edition*

**Table S2.** The final 89 feature list including the extended features, where the bold fonts indicate the extended feature.

| **No** | **Features** | | **No** | **Features** | |
| --- | --- | --- | --- | --- | --- |
| 1 | Age (year) | | 54 | Intent of treatment | |
| 2 | Gender | | 55 | Past history of gastric surgery | |
| 3 | Height (cm) | | 56 | Past history of endoscopic submucosal dissection | |
| 4 | Preoperative weight (kg) | | 57 | Operation method | |
| 5 | Postoperative one-year weight (kg) | | 58 | Lymph Node Dissection | |
| **6** | **Difference value of weight** | | 59 | Proximal resection margin (cm) | |
| **7** | **Difference percentage of weight** | | 60 | Distal resection margin (cm) | |
| 8 | Preoperative BMI | | 61 | Cancer stage | Ia |
| 9 | Postoperative one-year BMI (kg) | |  |  | Ib |
| **10** | **Difference value of BMI** | |  |  | IIa |
| **11** | **Difference percentage of BMI** | |  |  | IIb |
| 12 | Preoperative cholesterol (mg/dL) | |  |  | IIIa |
| 13 | Postoperative one-year cholesterol (mg/dL) | |  |  | IIIb |
| **14** | **Difference value of cholesterol** | |  |  | IIIc |
| **15** | **Difference percentage of cholesterol** | |  |  | IV |
| 16 | Preoperative hemoglobin (g/dL) | | 62 | Number of tumors | |
| 17 | Postoperative one-year hemoglobin (g/dL) | | 63 | Tumor size (cm) | |
| **18** | **Difference value of hemoglobin** | | 64 | Number of metastatic lymph nodes | |
| **19** | **Difference percentage of hemoglobin** | | 65 | Number of retrieved lymph nodes | |
| 20 | Preoperative albumin (g/dL) | | 66 | Extranodal extension of metastatic lymph node (pathological findings) | |
| 21 | Postoperative one-year albumin (g/dL) | | 67 | Diameter of extranodal extension of metastatic lymph node (mm) | |
| **22** | **Difference value of albumin** | | 68 | Lymphovascular invasion | |
| **23** | **Difference percentage of albumin** | | 69 | T stage | |
| 24 | Preoperative protein (g/dL) | | 70 | N stage | |
| 25 | Postoperative one-year protein (g/dL) | | **71** | **Perineural invasion** | **Negative** |
| **26** | **Difference value of protein** | |  |  | **Positive** |
| **27** | **Difference percentage of protein** | |  |  | **Not evaluated** |
| 28 | Preoperative nutritional risk index | | **72** | **Gross appearance of advanced gastric cancer (AGC)** | **Borrmann type 1** |
| 29 | Postoperative one-year nutritional risk index | |  |  | **Borrmann type 2** |
| **30** | **Difference value of NRI** | |  |  | **Borrmann type 3** |
| **31** | **Difference percentage of NRI** | |  |  | **Borrmann type 4** |
| 32 | Preoperative subcutaneous fat area (cm^2^) | |  |  | **Borrmann type 5** |
| 33 | Postoperative one-year subcutaneous fat area (cm^2^) | | **73** | **Gross appearance of early gastric cancer (Type 1 to 3)** | **Type I** |
| **34** | **Difference value of subcutaneous fat area** | |  |  | **Type II** |
| **35** | **Difference percentage subcutaneous fat area** | |  |  | **Type III** |
| 36 | Preoperative visceral fat area (cm^2^) | | **74** | **Tumor histology** | **Papillary adenocarcinoma** |
| 37 | Postoperative one-year visceral fat area (cm^2^) | |  |  | **Well-differentiated tubular adenocarcinoma)** |
| **38** | **Difference value of visceral fat area** | |  |  | **Moderately-differentiated tubular adenocarcinoma** |
| **39** | **Difference percentage visceral fat area** | |  |  | **Poorly-differentiated tubular adenocarcinoma** |
| 40 | Preoperative skeletal muscle area (cm^2^) | |  |  | **Signet-ring cell carcinoma** |
| 41 | Postoperative one-year skeletal muscle area (cm^2^) | |  |  | **Mucinous adenocarcinoma** |
| **42** | **Difference value of skeletal muscle area** | |  |  | **Others** |
| **43** | **Difference percentage skeletal muscle area** | | **75** | **Lauren Classification** | **Intestinal** |
| 44 | Preoperative skeletal muscle index (cm^2^/m^2^) | |  |  | **Diffuse** |
| 45 | Postoperative one-year skeletal muscle index (cm^2^/m^2^) | |  |  | **Mixed** |
| **46** | **Difference value of skeletal muscle index** | |  |  | **Indeterminate** |
| **47** | **Difference percentage skeletal muscle index** | | **76** | **Tumor location** | **Upper third** |
| 48 | Preoperative skeletal muscle index | | **77** |  | **Middle third** |
| 49 | Postoperative one-year skeletal muscle index | | **78** |  | **Lower third** |
| 50 | **Difference value of skeletal muscle index** | | **79** | **Comorbidity** | **Diabetes mellitus** |
| 51 | **Difference percentage of skeletal muscle index** | | **80** |  | **Hypertension** |
| 52 | **Type of surgery operation** | **Total gastrectomy** | **81** |  | **Chronic viral hepatitis** |
|  |  | **Distal gastrectomy** | **82** |  | **Liver cirrhosis** |
|  |  | **Other gastrectomy** | **83** |  | **Tuberculosis** |
| 53 | **Type of anastomosis** | **Gastroduodenostomy** | **84** |  | **Myocardial infarction** |
|  |  | **Roux-en-Y gastrojejunostomy** | **85** |  | **Cerebrovascular accident** |
|  |  | **Gastrojejunostomy without jejunojejunostomy** | **86** |  | **Valvular heart disease** |
|  |  | **Gastrojejunostomy with jejunojejunostomy** | **87** |  | **Chronic obstructive pulmonary disease** |
|  |  | **Total gastrectomy** | **88** |  | **Asthma** |
|  |  | **Others** | **89** |  | **Chronic renal failure** |

**Table S3.** Dataset summaries for training, internal validation, and external validation.

| **Data sources / Datasets** | | **Survived** | **Deceased** | **Total** |
| --- | --- | --- | --- | --- |
| AMC | Training | 3,079 (80%) | 141 (80%) | 3,220 (80%) |
|  | Internal validation | 770 (20%) | 35 (20%) | 805 (20%) |
|  | Total | 3,849 (100%) | 176 (100%) | 4,025 (100%) |
| AUH | External validation | 567 (100%) | 23 (100%) | 590 (100%) |

**Table S4.** Missing data rates for each feature according to the survived and deceased groups.

| **No** | **Characteristics** | **Description** | **AMC** | | | **AUH** | | |
| --- | --- | --- | --- | --- | --- | --- | --- | --- |
|  |  |  | **Survived**  **(%)** | **Deceased**  **(%)** | **Total**  **(%)** | **Survived**  **(%)** | **Deceased**  **(%)** | **Total**  **(%)** |
| **Demographic variables** | | | | | | | | |
| 1 | Age at operation (year) | | 0.0 | 0.0 | 0.0 | 0.0 | 0.0 | 0.0 |
| 2 | Gender | Male | 0.0 | 0.0 | 0.0 | 0.0 | 0.0 | 0.0 |
|  |  | Female | 0.0 | 0.0 |  | 0.0 | 0.0 |  |
| **Physical indices** | | | | | | | | |
| 3 | Height (cm) | | 0.2 | 0.0 | 0.2 | 0.0 | 0.0 | 0.0 |
| 4 | Preoperative weight (kg) | | 0.1 | 0.0 | 0.1 | 0.0 | 0.0 | 0.0 |
| 5 | Postoperative one-year weight (kg) | | 51.0 | 43.2 | 50.7 | 0.0 | 0.0 | 0.0 |
| 6 | Preoperative BMI | | 0.2 | 0.0 | 0.2 | 0.0 | 0.0 | 0.0 |
| 7 | Postoperative one-year BMI (kg) | | 51.2 | 43.2 | 50.8 | 0.0 | 0.0 | 0.0 |
| **Laboratory results** | | | | | | | | |
| 8 | Preoperative cholesterol (mg/dL) | | 0.0 | 0.0 | 0.0 | 0.0 | 0.0 | 0.0 |
| 9 | Postoperative one-year cholesterol (mg/dL) | | 3.4 | 3.4 | 3.4 | 0.0 | 0.0 | 0.0 |
| 10 | Preoperative hemoglobin (g/dL) | | 0.0 | 0.0 | 0.0 | 0.2 | 0.0 | 0.2 |
| 11 | Postoperative one-year hemoglobin (g/dL) | | 1.8 | 5.1 | 1.9 | 0.4 | 0.0 | 0.3 |
| 12 | Preoperative albumin (g/dL) | | 0.0 | 0.0 | 0.0 | 0.0 | 0.0 | 0.0 |
| 13 | Postoperative one-year albumin (g/dL) | | 3.4 | 3.4 | 3.4 | 0.2 | 0.0 | 0.2 |
| 14 | Preoperative protein (g/dL) | | 0.0 | 0.0 | 0.0 | - | - | - |
| 15 | Postoperative one-year protein (g/dL) | | 3.4 | 3.4 | 3.4 | - | - | - |
| **Nutritional index** | | | | | | | | |
| 16 | Preoperative nutritional risk index | | 0.1 | 0.0 | 0.1 | 0.0 | 0.0 | 0.0 |
| 17 | Postoperative one-year nutritional risk index | | 54.3 | 46.6 | 53.9 | 0.2 | 0.0 | 0.2 |
| **Body morphometry variables with fat/muscle indices** | | | | | | | | |
| 18 | Preoperative subcutaneous fat area (cm2) | | 74.5 | 69.3 | 74.2 | 0.0 | 0.0 | 0.0 |
| 19 | Postoperative one-year subcutaneous fat area (cm2) | | 70.2 | 61.4 | 69.8 | 0.0 | 0.0 | 0.0 |
| 20 | Preoperative visceral fat area (cm2) | | 74.5 | 69.3 | 74.2 | 0.0 | 0.0 | 0.0 |
| 21 | Postoperative one-year visceral fat area (cm2) | | 70.2 | 61.4 | 69.8 | 0.0 | 0.0 | 0.0 |
| 22 | Preoperative skeletal muscle area (cm2) | | 74.5 | 69.3 | 74.2 | 0.0 | 0.0 | 0.0 |
| 23 | Postoperative one-year skeletal muscle area (cm2) | | 70.2 | 61.4 | 69.8 | 0.0 | 0.0 | 0.0 |
| 24 | Preoperative skeletal muscle index (cm2/m2) | | 74.5 | 69.3 | 74.2 | 0.0 | 0.0 | 0.0 |
| 25 | Postoperative one-year skeletal muscle index (cm2/m2) | | 70.2 | 61.4 | 69.8 | 0.0 | 0.0 | 0.0 |
| 26 | Preoperative skeletal muscle index | | 74.5 | 69.3 | 74.2 | 0.0 | 0.0 | 0.0 |
| 27 | Postoperative one-year skeletal muscle index | | 78.6 | 71.0 | 78.2 | 0.0 | 0.0 | 0.0 |
| **Surgery-related variables** | | | | | | | | |
| 28 | Type of surgery operation | Total gastrectomy | 0.1 | 0.6 | 0.1 | 0.2 | 0.0 | 0.2 |
|  |  | Partial gastrectomy | 0.1 | 0.6 | 0.1 | 0.2 | 0.0 | 0.2 |
|  |  | Other gastrectomy | 0.1 | 0.6 | 0.1 | 0.2 | 0.0 | 0.2 |
| 29 | Type of anastomosis | Gastroduodenostomy | 9.4 | 14.2 | 9.6 | 0.2 | 0.0 | 0.2 |
|  |  | Roux en Y gastrojejunostomy | 9.4 | 14.2 | 9.6 | 0.2 | 0.0 | 0.2 |
|  |  | Gastrojejunostomy without jejunojejunostomy | 18.8 | 28.4 | 19.2 | 0.4 | 0.0 | 0.4 |
|  |  | Gastrojejunostomy with jejunojejunostomy | 9.4 | 14.2 | 9.6 | 0.2 | 0.0 | 0.2 |
|  |  | Roux-en-Y | 9.4 | 14.2 | 9.6 | 0.2 | 0.0 | 0.2 |
|  |  | Others | 9.4 | 14.2 | 9.6 | 0.2 | 0.0 | 0.2 |
| 30 | Intent of treatment | | 0.0 | 0.0 | 0.0 | - | - | - |
| 31 | Past history of gastric surgery | | 9.1 | 14.2 | 9.3 | - | - | - |
| 32 | Past history of endoscopic submucosal dissection | | 9.0 | 14.2 | 9.3 | - | - | - |
| 33 | Operation method | | 3.5 | 5.7 | 3.6 | - | - | - |
| 34 | Lymph Node Dissection | | 4.3 | 7.4 | 4.4 | - | - | - |
| 35 | Proximal resection margin (cm) | | 0.5 | 1.7 | 0.6 | - | - | - |
| 36 | Distal resection margin (cm) | | 0.5 | 1.7 | 0.6 | - | - | - |
| **Pathologic variables** | | | | | | | | |
| 37 | Cancer stage (Ia, Ib, IIa, IIb, IIIa, IIIb, IIIc, IV) | | 0.0 | 0.0 | 0.0 | 0.0 | 0.0 | 0.0 |
| 38 | Number of tumors | | 9.2 | 15.3 | 9.4 | - | - | - |
| 39 | Tumor size (cm) | | 0.2 | 0.0 | 0.2 | - | - | - |
| 40 | Number of metastatic lymph nodes | | 0.1 | 0.6 | 0.1 | - | - | - |
| 41 | Number of retrieved lymph nodes | | 0.1 | 0.6 | 0.1 | - | - | - |
| 42 | Extranodal extension of metastatic lymph node (pathological findings) | | 75.4 | 64.2 | 74.9 | - | - | - |
| 43 | Diameter of extranodal extension of metastatic lymph node (mm) | | 96.4 | 92.1 | 96.2 | - | - | - |
| 44 | Lymphovascular invasion | | 0.7 | 0.6 | 0.7 | - | - | - |
| 45 | T stage | | 0.2 | 0.6 | 0.2 | - | - | - |
| 46 | N stage | | 0.1 | 0.6 | 0.1 | - | - | - |
| 47 | Perineural invasion | Negative | 1.8 | 2.8 | 1.8 | - | - | - |
|  |  | Positive | 1.8 | 2.8 | 1.8 | - | - | - |
|  |  | Not evaluated | 1.8 | 2.8 | 1.8 | - | - | - |
| 48 | Gross appearance of advanced gastric cancer (AGC) | Borrmann type 1 | 65.6 | 44.9 | 64.7 | - | - | - |
|  |  | Borrmann type 2 | 65.6 | 44.9 | 64.7 | - | - | - |
|  |  | Borrmann type 3 | 65.6 | 44.9 | 64.7 | - | - | - |
|  |  | Borrmann type 4 | 65.6 | 44.9 | 64.7 | - | - | - |
|  |  | Borrmann type 5 | 65.6 | 44.9 | 64.7 | - | - | - |
| 49 | Gross appearance of early gastric cancer  (Type 1 to 3) | Type I | 38.5 | 60.8 | 39.5 | - | - | - |
|  |  | Type II | 38.5 | 60.8 | 39.5 | - | - | - |
|  |  | Type III | 38.5 | 60.8 | 39.5 | - | - | - |
| 50 | Tumor histology | Papillary adenocarcinoma | 10.2 | 16.5 | 10.4 | - | - | - |
|  |  | Well-differentiated tubular adenocarcinoma) | 10.2 | 16.5 | 10.4 | - | - | - |
|  |  | Moderately-differentiated tubular adenocarcinoma | 10.2 | 16.5 | 10.4 | - | - | - |
|  |  | Poorly-differentiated tubular adenocarcinoma | 10.2 | 16.5 | 10.4 | - | - | - |
|  |  | Signet-ring cell carcinoma | 10.2 | 16.5 | 10.4 | - | - | - |
|  |  | Mucinous adenocarcinoma | 10.2 | 16.5 | 10.4 | - | - | - |
|  |  | Others | 10.2 | 16.5 | 10.4 | - | - | - |
| 51 | Lauren Classification | Intestinal | 7.7 | 10.2 | 7.8 | - | - | - |
|  |  | Diffuse | 7.7 | 10.2 | 7.8 | - | - | - |
|  |  | Mixed | 7.7 | 10.2 | 7.8 | - | - | - |
|  |  | Indeterminate | 7.7 | 10.2 | 7.8 | - | - | - |
| 52 | Tumor location | Upper third | 0.6 | 0.6 | 0.6 | - | - | - |
| 53 |  | Middle third | 0.6 | 0.6 | 0.6 | - | - | - |
| 54 |  | Lower third | 0.6 | 0.6 | 0.6 | - | - | - |
| **Comorbidities** | | | | | | | | |
| 55 | Diabetes mellitus | | 64.3 | 47.7 | 63.6 | - | - | - |
| 56 | Hypertension | | 64.3 | 47.7 | 63.6 | - | - | - |
| 57 | Chronic active hepatitis | | 64.3 | 47.7 | 63.6 | - | - | - |
| 58 | Liver cirrhosis | | 64.3 | 47.7 | 63.6 | - | - | - |
| 59 | Tuberculosis | | 64.3 | 47.7 | 63.6 | - | - | - |
| 60 | Myocardial infarction | | 64.3 | 47.7 | 63.6 | - | - | - |
| 61 | Cerebrovascular accident | | 64.3 | 47.7 | 63.6 | - | - | - |
| 62 | Valvular heart disease | | 64.3 | 47.7 | 63.6 | - | - | - |
| 63 | Chronic obstructive pulmonary disease | | 64.3 | 47.7 | 63.6 | - | - | - |
| 64 | Asthma | | 64.3 | 47.7 | 63.6 | - | - | - |
| 65 | Chronic renal failure | | 64.3 | 47.7 | 63.6 | - | - | - |
